# Blood component-associated acute transfusion reactions in pediatric patients: experience of a tertiary care hospital

**DOI:** 10.55730/1300-0144.5869

**Published:** 2024-07-16

**Authors:** Zeliha GÜZELKÜÇÜK, Dilek GÜRLEK GÖKÇEBAY, Turan BAYHAN, İkbal OK BOZKAYA, Özlem ARMAN BİLİR, Vildan KOŞAN ÇULHA, Melek IŞIK, Ayca KOCA YOZGAT, Hüsniye Neşe YARALI, Namık Yaşar ÖZBEK

**Affiliations:** 1 Department of Pediatrics, Division of Pediatric Hematology Oncology, Ankara Bilkent City Hospital, Ankara, Turkiye; 2 Department of Pediatrics, Division of Pediatric Hematology Oncology, Ankara Bilkent City Hospital, University of Health Sciences, Ankara, Turkiye; 3 Department of Pediatrics, Division of Pediatric Hematology Oncology, Faculty of Medicine, Ankara Yıldırım Beyazıt University, Ankara, Turkiye

**Keywords:** Blood, transfusion, reaction, platelet, granulocyte, children

## Abstract

**Background/aim:**

The transfusion of blood products is a life-saving clinical practice in patients with bleeding, hemoglobinopathy, and cancer. It was aimed herein to analyze the frequency and types of blood component-related acute transfusion reactions (ATRs) in pediatric patients.

**Materials and methods:**

This retrospective study was conducted at a tertiary care academic pediatric hospital.

**Results:**

During the study period, 30,811 transfusions were administered to 25,448 patients. There were 103 ATRs detected in 81 patients (0.33%; 3.34 reactions per 1000 transfusions, mean age 8.3 ± 5.98 years, 36 females and 45 males). All the reactions were observed within an average of 4 h after the transfusion began. The most common ATRs were allergic reactions (79; 76.6%) and febrile nonhemolytic transfusion reactions (12; 11.6%). All the allergic transfusion reactions occurred within the first hour after the start of the transfusion. Granulocyte concentrates were the blood component associated with the highest ATR rate (2.1%).

**Conclusion:**

Within our hospital, pediatric hematology-oncology wards and the stem cell transplantation unit had the most frequent ATR reports; therefore, when transfusions are carried out, increased attention should be given to these units. Educating health staff about the adverse effects of transfusion therapy should increase the awareness and reporting of ATRs in children.

## 1. Introduction

The transfusion of blood products is a life-saving clinical practice in patients with bleeding, hemoglobinopathy, and cancer [1]. However, it brings several adverse effects, ranging from mild to life-threatening reactions [2]. These reactions may be unpredictable despite significant advances in transfusion practice [3,4]. Transfusion reactions may be acute or delayed due to the time of occurrence. Hemolytic transfusion reactions, febrile nonhemolytic transfusion reactions (FNHTRs), allergic reactions, transfusion-related acute lung injury (TRALI), transfusion-related circulatory overload (TACO), hypotensive reactions, hypothermia and metabolic disorders are defined as acute transfusion reactions (ATRs) [5].

Knowledge on transfusion reactions in children is limited compared to that for adults [6]. Most guidelines for transfusion reactions are based on data from adult patients [7]. In a previous study conducted on children, factors such as the type of blood component, age, patient comorbidity, and multiple transfusions were associated with transfusion reactions [8]. However, the pathophysiology of transfusion reactions in the pediatric population is not fully understood [6]. In this study, it was aimed to analyze the frequency and types of blood product-related ATRs in pediatric patients in a children’s hospital where approximately 17,500 transfusions are carried out annually.

## 2. Material and methods

### 2.1. Patients

This retrospective study was conducted at a tertiary care academic pediatric hospital. Inpatient services include departments of pediatric hematology/oncology, hematopoietic stem cell transplantation, neonatal and pediatric intensive care, burn, trauma, and pediatric surgery. Children between 0 and 18 years of age who had received a blood component transfusion between January 2020 and September 2021 were included in the study. Children who had developed a transfusion-related reaction during and/or within the first 24 h after cessation of the transfusion were accepted as having an ATR. Detailed data concerning the transfusion procedures and ATRs were obtained from the patients’ records compiled by the hemovigilance unit. The age, sex, department of hospitalization, blood component types, additional processing (irradiation, filtration, etc.), previous transfusion history, and ATR types were recorded. Patients who received intravenous immunoglobulin, coagulation factor concentrate, albumin, or antithymocyte globulin were not included in the study.

### 2.2. Definitions

A transfusion reaction was defined as an undesirable response or effect in a patient that is temporally associated with the administration of blood components in accordance with the National Hemovigilance Guideline [9]. An ATR was defined as a reaction that occurred within the first 24 h after the transfusion began. In Türkiye, most of the blood products are produced and distributed by the Turkish Red Crescent and the others are by temporary regional blood centers except for the granulocyte concentrates (GCs). All the cellular blood components [red blood cell concentrates (RBCs), pooled platelet concentrates (PPCs) and apheresis platelet concentrates (APCs)] were prestorage filtered for leuko-reduction by the Turkish Red Crescent Regional Blood Bank. Moreover, nucleic acid tests were performed for all the blood products by the Turkish Red Crescent. GCs were prepared from a single donor with granulocyte-colony stimulating factor and dexamethasone stimulation 12 h before collection in our pediatric apheresis unit. They usually contained more than 1.0 × 10^10^ granulocytes suspended in 200–300 mL of plasma. Cellular blood components of immune-compromised patients were irradiated with 25 Gy gamma irradiation in our transfusion center.

During blood component transfusion, vital signs of the patient were recorded every 15 min by a clinical nurse in our hospital. In the case of a reaction, the physician in-charge was notified and the relationship of the reaction to the transfusion is evaluated. Signs and symptoms related to transfusion reactions and imputability of the blood product concerned were evaluated in accordance with the definitions of the National Hemovigilance Guideline (grades 1–4) [9].

ATRs were classified as hemolytic reactions, FNHTRs, mild allergic reactions, anaphylactoid reactions, bacterial contamination, TRALI, TACO, transfusion-associated hypotension, hypothermia, citrate toxicity, and metabolic disorders. When the reaction was determined as transfusion-related, the severity and characteristics of the reaction, blood component type, the patient’s vital signs, and the treatment were recorded in the National Hemovigilance Registry System of the Ministry of Health [9].

### 2.3. Ethics committee approval

This study was approved by the local ethics committee (approval number: E2-21-969).

### 2.4. Statistical analysis

Analysis of the data was primarily descriptive, using standard deviations, ranges, and mean and median values. Categorical variables were analyzed using the χ^2^ test, and continuous variables were analyzed using the Student’s t test. p < 0.05 was considered statistically significant for all the analyses. IBM SPSS Statistics for Windows 21.0 (IBM Corp., Armonk, NY, USA) was used for all the statistical analyses.

## 3. Results

During the study period, 30,811 transfusions [13.797 (44.7%) RBC units; 6853 (22.2%) PPCs; 6812 (22.1%) FFPs; 1766 (5.7%) cryoprecipitates; 1298 (4.2%) APCs; 279 (0.9%) GCs; six COVID-19 immune plasma] were performed in 25,448 patients. In total, 103 (0.33%) ATRs were recorded in 81 patients. The frequency of ATRs was 3.34 reactions per 1000 transfusions. In seventy-seven (74.5%) of the 103 reactions, patients had a history of previous transfusion. Fifteen patients experienced more than one ATR (min–max number of reactions: 1–4). In 10 patients two reactions, in four patients three reactions, and in one patient five reactions occurred. Eighteen out of the 21 recurrent transfusion reactions were mild allergic reactions and the others were FNHTRs. In patients with recurrent mild allergic reactions, washing of the blood components could not be performed for technical reasons. Premedication (antihistamine and antipyretic drugs) was given to prevent transfusion reactions in patients who had a previous transfusion reaction. Seventy-eight (75.7%) of the ATRs were observed with irradiated blood components. Imputability of the reactions were graded as, 1 in 77 reactions, and 2 in 26 reactions.

The mean age of the patients who developed ATRs was 8.3 ± 5.98 years (36 females, 45 males). All the reactions occurred within 4 h from the start of the transfusion, whereas all allergic transfusion reactions occurred within the first hour. Grade 1–2 ATRs were observed in 86 transfusions and grade 3 ATRs were observed in 17 transfusions. The patient who had grade 3 was diagnosed with TRALI. Seventy-nine (76.6%) of the ATRs were allergic reactions, 12 (11.6%) were FNHTRs, one (0.97%) was a hypotensive reaction, and another one (0.97%) was TRALI. Ten (9.7%) reactions could not be classified, with symptoms including nausea, dizziness, and weakness. Allergic transfusion reactions were mostly mild in 63 patients (79.7%), and anaphylactic reactions were observed in 16 patients (20.3%). The most common presentations of allergic reaction were rash (78.2%), followed by respiratory system symptoms including cough and shortness of breath (16.5%). Angioedema, wheezing, cough, abdominal pain, and vomiting were also observed in patients diagnosed with anaphylaxis. The symptoms during the ATRs are shown in Table 1. Patients with mild allergic reactions were treated with antihistamines, while patients with anaphylaxis were treated with adrenaline, antihistamines and corticosteroids. FNHTRs were successfully managed with antipyretic therapy. Supportive treatment was given according to the severity of the symptoms in patients with hypotensive transfusion reactions and unspecified reactions. The patient with TRALI was a 13-year-old male who had dyspnea and decreased oxygen saturation 1 h after a RBC transfusion. The chest X-ray showed diffuse bilateral pulmonary infiltration. He was treated with oxygen support and recovered after three days. None of the patients with ATRs died.

The frequency of transfusion reactions for each blood component are shown in Table 2. Although GCs were used less frequently compared to the other blood components, adverse reactions were more frequently observed with this product (2.1%). Mild allergic reactions (three), anaphylactic reactions (two) and FNHTR (one) were seen in patients receiving GCs. One-third of the blood component transfusions were performed in the hematology-oncology departments and the stem cell transplantation unit; therefore, these are where the ATRs most commonly occurred.

## 4. Discussion

Although data on transfusion reactions in childhood is scarce, previous studies revealed that transfusion reactions in children are about twice as common compared to those in adults [6,8,10]. The incidence of ATRs was 0.33% and approximately three-quarters of the ATRs were allergic reactions in the current study. Despite the fact that GCs are less commonly used blood products, the frequency of ATRs was remarkably higher than those for the other products (2.15% reactions with GCs vs. 0.33% for all the blood components). Nevertheless, studies on transfusion reactions lack data on the rates and features of reactions due to GCs, which may be related to the unavailability of GCs in all centers [4,7,8,10]. We believe that evaluation of the characteristics of ATRs in the pediatric population is important for the determination of standard clinical approaches.

ATRs vary in type and frequency in children and adults [5]. According to the UK 2016 Serious Hazards of Transfusion (SHOT) report, 1.8 million blood components were transfused and 403 clinical adverse events, mostly acute allergic or febrile reactions, were recorded [11]. A study from China revealed hemovigilance data of a five-year period and the incidence of ATRs in children was 0.034%, which was very low compared to the present study (0.33%) [12]. Another study from Türkiye disclosed that the frequency of ATRs was 0.09% in a group consisting of adult and pediatric patients aged one month–85 years [13]. However, in a study including only children in intensive care units, a frequency of ATRs as high as 1.6% in a two year period was obtained [14]. In these mentioned studies, there were many differences involving the patients’ age, underlying diseases, and study duration. Therefore, the frequency of ATRs differed greatly from each other. Hence, multicenter studies are needed in order to reach a final conclusion.

The results of a previous study were in line with those obtained herein in terms of allergic reactions. In their study, Kulhas Celik et al. [15] evaluated 157 children who developed 125 allergic transfusion reactions and found that 79% of the reactions were minor allergic reactions and 21% were anaphylactoid reactions. It was also reported that the most common blood components associated with allergic transfusion reactions were apheresis and single-donor PCs (34.5%) [15,16]. In the present study, 44% of the allergic transfusion reactions occurred with apheresis and PPCs. Although PPCs were usually obtained from four donors in the present study, it is noteworthy that the cumulative incidence of allergic reactions was higher in patients receiving APCs compared to those receiving PPCs. A recent study revealed that the most important feature of ATRs related to PCs was the number of platelets given per transfusion [17]. In addition, it has been shown that the incidence of allergic reactions was lower with pooled platelets suspended in platelet additive solution compared to those with APCs [18]. However, in the current study, the number of platelets in the PPCs could not be measured; therefore, comparing the number of platelets in the PPCs and APCs was not possible. No life-threatening allergic transfusion reactions were observed. Moreover, since all the allergic transfusion reactions occurred within the first hour after the transfusion began, we believe children should be monitored closely in the early phase of transfusion for allergic reactions.

In a recent study, the second most frequently reported ATR following allergic reactions was FNHTR, as in the current study [19]. In that study, 24 FNHTR occurred during 2509 transfusions (2.9%) in a pediatric intensive care unit [14]. In the present study, this rate was lower (0.072%). FNHTRs can occur due to factors related to the donor, blood component, and/or recipient. Moreover, diagnosis could be difficult in children because of underlying diseases that may cause fever [20]. It was shown that leuko-filtration reduces the frequency of this reaction [5]. All the cellular blood products that were transfused herein were leuko-filtered before storage and most of them were irradiated. Hence, the less frequent occurrence of FNHTR in the current study population could have been due to the use of leuko-depleted cellular blood components. A recent study revealed that a significant increase in the frequency of FNHTR or TRALI was associated with prolonged storage time of apheresis/irradiated PCs [16]. It was suggested that an increase of cytokines in blood products during storage could contribute to increased frequency of ATRs [16]. In the present study, the storage time for the cellular blood was five and 42 days for the PCs and RBCs, respectively. In our hospital, blood products are used until the end of the expiration date. In the current study, the relationship between the storage time and frequency of ATRs could not be evaluated.

GC transfusions have been reported to be associated with minor ATRs [21]. Nevertheless, they may cause serious life-threatening pulmonary complications, especially in patients with preexisting pneumonia. Severe pulmonary reactions generally occur in patients with preexisting respiratory dysfunction due to the sequestration of transfused cells in the pulmonary vascular system. In addition, ATRs associated with GCs have also been reported in patients who were alloimmunized against human leukocyte antigens [22,23]. Premedication with acetaminophen, antihistamines, or steroids is recommended for the prevention of ATRs due to GC transfusions [21,22]. The current study revealed a higher cumulative rate of ATRs (2.15%) associated with GCs used in pediatric cancer or stem cell transplant patients despite premedication. In the present study, six ATRs (three mild allergic reactions, two anaphylactic reactions, and one FNHTR) were found during 279 GC transfusions. Since data concerning ATRs due to GC transfusion are scarce, further studies on adverse events in GC transfusions with a larger patient group are necessary.

The annual SHOT report in 2018 declared that pulmonary complications related to transfusions caused major morbidity in the UK [24]. These are TRALI, TACO, and transfusion-associated dyspnea (TAD), which are often overlapping diagnoses. In adult patients, TRALI is a rare but important cause of transfusion-related mortality [25]. TRALI should always be kept in mind in patients who develop respiratory symptoms within the first 6 h of transfusion [25]. In the current study, only one patient was diagnosed with TRALI after RBC transfusion. Another reaction involving symptoms of the respiratory system is TACO [5]. TACO is the most commonly reported cause of transfusion-related mortality [24]. There is an urgent need to determine standard and usable criteria for the diagnosis of TACO, particularly in young children [25,26]. The diagnosis of TRALI or TACO may have been overlooked in the present study, and we believe that the incidence would increase through continuing education to increase awareness of healthcare professionals to these pulmonary complications.

It is well-known that patients who had a previous transfusion history, and those with a previously reported transfusion reaction have an increased risk of developing ATRs [8]. In our hospital, most of the reactions were reported in patients from the hematology-oncology wards and stem cell transplantation unit, in which transfusion is a frequent event. A history of previous transfusion was present in about three-quarters of the patients with ATRs and more than one reaction was reported in 15 patients. Because the severity of the reactions was mild, and due to technical problems, washing of the blood components could not be performed in patients with recurrent allergic reactions. However, knowledge of the underlying mechanism is limited, and it has been suggested that patients with ATRs should be evaluated for preventive measures [8].

The limitations of the study were related to its retrospective design. However, since this study included only the data of pediatric patients and in particular, of hematology-oncology patients, we believe that the findings are valuable.

## Conclusion

All the allergic transfusion reactions occurred within the first hour after the transfusion began and there was a need for careful follow-up during this phase. GCs were the blood component associated with the highest ATR rate. Within our children’s hospital, the pediatric hematology-oncology wards and the stem cell transplantation unit had the most frequent ATR reports. Hence, increased attention should be given when transfusions carried out in these units. Implementation of a hemovigilance system and education for health staff on transfusion therapy should increase awareness and reporting of ATRs in children.

## Figures and Tables

**Table 1:** Symptoms during ATRs.

Symptoms	Number	%
Rash	80	78.2
Respiratory system symptoms	17	16.5
Fever	13	12.6
Dizziness-weakness-nausea	10	9.7
Chills	5	4.8
Abdominal pain	4	3.8
Vomiting	4	3.8

**Table 2:** The frequency of transfusion reactions in terms of blood products.

	RBC (n, %)	PPC (n, %)	FFP (n, %)	APC (n, %)	Cryoprecipitate (n, %)	GC (n, %)
Mild Allergic	19 (54.2)	16 (61.5)	16 (80)	8 (53.3)	1 (100)	3 (50)
FNHTR	10 (28.5)	1 (3.85)				1 (16.6)
Unspecified	4 (11.4)	3 (11.5)	2 (10)	1 (6.6)		
Anaphylaxis	1 (2.8)	5 (19.2)	2 (10)	6 (40)		
TRALI	1 (2.8)					
Hypotension		1 (3.85)				
Total	35 (0.25)	26 (0.37)	20 (0.29)	15(1.15)	1(0.05)	6 (2.10)

FNHTR; febrile nonhemolytic transfusion reactions, TRALI; transfusion-related acute lung injuries, RBC; red blood cell concentrate, PPC; pooled platelet concentrate, FFP; fresh frozen plasma APC; apheresis platelet concentrate, GS; granulocyte suspension.
